# Bioturbation by black soldier fly larvae—Rapid soil formation with burial of ceramic artifacts

**DOI:** 10.1371/journal.pone.0252032

**Published:** 2021-06-02

**Authors:** Juan M. Orozco-Ortiz, Sara L. Bauke, Christian Borgemeister, Eva Lehndorff, Wulf Amelung

**Affiliations:** 1 Institute of Crop Science and Resource Conservation (INRES), University of Bonn, Bonn, Germany; 2 Center for Development Research (ZEF), University of Bonn, Bonn, Germany; 3 Soil Ecology, Bayreuth University, Bayreuth, Germany; Feroze Gandhi Degree College, INDIA

## Abstract

Bioturbation involves the incorporation of residues from the surface soil into the subsoil; however, common small soil ‘bioengineers’, such as earthworms or termites, are unlikely to transport human artifacts to deeper soil horizons. However, such artifacts occur in the deeper soil horizons within Amazonian Anthrosols (Terra Preta). Here we test the assumption that such tasks could be carried out by fly larvae, which could thus play a crucial role in waste decomposition and associated soil mixing under tropical conditions. We performed two greenhouse experiments with sandy substrate covered with layers of organic waste, ceramic fragments, and black soldier fly larvae (BSFL) (*Hermetia illucens* (L.) (Dipt.: Stratiomyidae)). We used in-situ images to assess the rate of bioturbation by BSFL, and then designed our main study to observe waste dissipation (reduction of organic carbon and phosphorus contents from waste model trials with and without charcoal) as related to larval-induced changes in soil properties. We found that the bioturbation of macroinvertebrates like BSFL was able to bury even large (> 5 cm) ceramic fragments within hours, which coincided with high soil growth rates (0.5 cm h^-1^). The sandy soil was subsequently heavily enriched with organic matter and phosphorus originating from organic waste. We conclude that BSFL, and possibly other fly species, are important, previously overlooked soil ‘bioengineers’, which may even contribute to the burial of artifacts in Anthrosols and other terrestrial waste dumps.

## Introduction

Anthrosols are soils that have been transformed, influenced, or created by human activities [1]. These soils are found in different places around the world [2–4]. Special attention has been paid to Amazonian Anthrosols in the past decades [5], which occur in and around ancient tropical settlements [6]. These soils that presumably formed in pre-Columbian middens are also known as Archaeological Black Earths (ABE), Amazonian Dark Earths (ADE), or Terra Preta (Black soils in Portuguese), because they generally have a darker coloration, higher fertility than surrounding soils, and show the presence of potsherds and charcoal in different amounts [7]. Most likely, Terra Preta (TP) soils are the product of continuous waste (i.e., food scrap, kitchen waste, charcoal, feces, etc.) discard on top of typically highly weathered, infertile Amazonian soils [8, 9]. However, it has remained unclear:

- Which organisms were involved in waste turnover and bioturbation?
- How long did the organic waste reside on top of the soil before its incorporation?
- How could the potsherds and other prominent artifacts have been buried deep into subsoil in an Amazonian context?

Here, we aim to contribute to a better understanding of the above open questions.

Soil bioturbation occurs significantly faster than soil production via rock weathering and, in all likelihood much, faster than many other pedogenic processes [10]. This study was based on the working hypothesis that beneficial fly species, and specifically black soldier fly larvae (BSFL) (*Hermetia illucens* [L.] [Dipt.: Stratiomyidae]), play a crucial role in the formation of Amazonian Anthrosols. Arguments in favor of our hypothesis are i) Amazonian climatic conditions are ideal for black soldier flies (BSF), ii) the females are attracted to a wide variety of decomposing waste [11–13], and iii) BSFL consume domestic waste after a few weeks of continuous waste disposal [14]. Hence, the fact that indigenous populations were discarding ceramics and organic waste at the same time could have led to preferential colonization of such materials, and by consequence, to a faster and more efficient waste turnover, bioturbation, and subsequent soil formation.

Soil formation, in the strict sense, is an integration of specific formation processes which can be classified, among others, according to formation times (also known as characteristic times or rates) with rapid (10^1−2^ years), medium (10^3−4^ years), and slow rates (10^5−6^ years) [15]. When biomaterials are added and mixed into soil, soil volume increases, resulting in a particular soil formation process that we denominate here as soil growth. Smith [16] suggested a ratio of 1 cm of incremental TP growth for every ten years. Based on evidence from three TP sites, Neves et al. [17] proposed an even faster TP formation process and postulated that TP formation “is not so much dependent on the length of time alone, but rather more on the intensity of occupation over time, allowing for the constant input of organic matter and nutrients to the soil” [17, p: 43].

We suggest that TP soil growth can be categorized as a tropical example of what could be interpreted as biomantle, i.e., the mixed upper layers of soil formed by biota [18]. A faunalmantle would be a more precise term to describe TP formation if we consider it as the result of faunalturbation (the action of animals) [19, 20]. Yet, to the best of our knowledge, bioturbating flies have not been considered as organisms contributing to TP pedogenesis or other soil groups.

Cunha et al. [21] proposed that earthworms contribute to TP formation, but this heavily depends on the environmental conditions during early TP genesis. Indeed, earthworms might have colonized TP after the discarded domestic waste had turned into a soil-like stable material; however, they would not survive the low pH and chemical compounds, such as alcohols and acids produced during the early stages of waste decomposition [22]. Furthermore, it appears questionable to what degree earthworms would have contributed to artifacts’ rapid burial, like ceramics. This latter argument also applies to termites, which are important ‘bioengineers’ in tropical soil and smaller than earthworms [23]. In contrast, BSFL can feed on and break down a wider variety of organic matter [13, 24] than earthworms [25] or other waste management strategies like composting [26]. BSFL are known to control pathogens present in waste [12, 27] and human feces [11] and to reduce the concentration of elements (i.e., total organic carbon (TOC), nitrogen (N), phosphorus (P)) present in high-nutrient materials like domestic wastes [28]. BSF have been proposed as an excellent environmental engineer to treat food waste [29, 30]. Studies suggest that BSF could sanitize organic waste, and its bioconversion by-product could be of use as a soil amendment [24, 31]. However, BSF’s inconspicuous ecosystem-bioengineer facet, particularly in the formation and bioturbation of Anthrosols, has not yet been studied.

Short-term methods to assess soil mixing have been used for many years [32, 33], recently including the vivid observation of macrofauna functioning in different environments [34]. These short-term methods allow for the quantification of soil growth, also referred to as Soil Production Rate (SPR). SPR or soil growth could also be considered as the rate at which the faunalmantle grows, similar to the production of casts in the case of earthworms [35]. Evidence of BFSL activity in soils may potentially also be derived from amino sugars, which have widely been used as an integrated marker for microbial necromass in soil, whereas respective contributions from living microorganisms are small [36–38]. Besides, these markers are relatively stable in time; Derrien and Amelung [39] assumed a half-life of 6–8 years. Hence, no significant changes in these marker concentrations can be considered by mere microbial performance within a short-term incubation study. A contribution of amino sugars from soil animals is usually neglected within these analyses [37]. However, the larvae’s cuticle could introduce amino sugars in a BSFL waste treatment system. While we did not find any report on larvae amino sugar contents, the occurrence of chitin in larvae cuticles [40, 41] should result in significant inputs of chitin-derived glucosamine. We combine such research with phosphorus pools analyses, thus providing knowledge on how this essential nutrient is mixed into the soil by larval bioturbation. For example, high amounts of calcium-bound phosphorus (Ca-P) found in TP have been linked to biogenic apatite formation from fish remains [42–44].

In combining these analyses, we aim to elucidate whether BSFL contribute to the early stages of organic waste decomposition and incorporation into the soil, as may have occurred during the formation of Amazonian Anthrosols, such as TP. We hypothesized that (i) changes in soil properties can occur already within a few hours when BSFL feed on organic wastes that are placed on top of poor artificial soils and that (ii) bioturbation of the soil matrix, including ceramics, may be mediated by BSFL more extremely and intensely than other organisms. To test these hypotheses, we applied BSFL and an organic waste model to sandy substrates in a greenhouse environment and discussed the results and implications for understanding TP formation.

## Materials and methods

We conducted one main experiment and one experiment for an illustrative video, both were done in the greenhouse using artificial soil and waste models with and without charcoal, as the latter is a common constituent in TP. These two waste models were further combined with BSFL and ceramic sherds.

The video documentation was performed to illustrate the process of larvae bioturbation and related waste decomposition (see S1 Video at https://osf.io/x35kn/), which helps understand the main experiment’s design and exhibits the ability of BSFL to remain inactive during days without food within the faunalmantle, but also to rapidly process waste containing charcoal. We used artificial soil in combination with an organic waste model (WM) and ceramics and prepared two treatments with and without BSFL (with only one replicate each).

The main experiment was conducted with 5 replicates each for six treatment combinations and one control (see Table 1). It was designed to quantify the influence of ceramics and larvae on waste turnover and nutrient incorporation into the artificial soil. If not mentioned in the following segments, the conditions for the two experiments did not differ. Temperature inside the greenhouse was kept at 26 ±1°C for both experiments.

**Table 1:** Experimental setup.

Treatment	Sand-Clay soil /g	Waste Model (Dog Food+Pig slurry)/g	Biochar/g	Ceramics/g	Larvae/g
**Absolute Control**	2503±7	0	0	0	0
**Waste Model (WM) (Control)**	2503±7	1674±5	0	0	0
**Waste Model with Charcoal (WMC) (Control)**	2503±7	1674±5	81±4	0	0
**WM + Ceramics Sherds**	2503±7	1674±5	0	238±3	0
**WMC + Ceramic Sherds**	2503±7	1674±5	81±4	238±3	0
**WM + Larvae + Ceramic Sherds**	2503±7	1674±5	0	238±3	134±10
**WMC + Larvae + Ceramic Sherds**	2503±7	1674±5	81±4	238±3	134±10

Five replicates (n = 5) for each of the main experiment’s treatments.

### Materials

The artificial soil used for the incubation tests was a sandy mineral substrate modified from Adani and Spagnol [45] and consisted of unfertilized, fresh, unused sand (Quarzwerke Alfter Witterschlick, Germany, particle size Ø = 0.4–0.8 mm), and clay (kaolinite like material for tile production, Röben Tonbaustoffe GmbH factory, Brüggen, Germany, ground and sieved to <1 mm). Sand and clay were mixed at a ratio of 9:1 (w/w). Plastic containers were filled with the soil and moistened with water to 40% (w/w) of the maximum water-holding capacity determined according to Alef and Nannipieri [46]. Water content was maintained by weighing the containers every fifth day and adding water until the initial weight was reached again. Sandy mineral substrates avoid contamination from soil organic matter (SOM) and have been previously used for TP greenhouse experiments [see 47].

To simulate pre-Columbian-like organic waste input to TP sites, we used an organic WM from a mixture of digested pig slurry and dog food [12]. The digested pig slurry was collected from the Frankenforst campus of the University of Bonn, Germany. It was then mixed with dog food (Pro Plan medium puppy, Purina, Germany) at a ratio of 4:1 (28±1.2% total solids). Commercially available barbecue charcoal (Weber Holzkohle, Germany; manually ground and sieved to < 2 mm) was used as a model for biochar. Barbecue charcoal has also been utilized in other studies to test effects of biochar in SOM on soil fertility [47], because it is produced at a temperature ≈ 400°C and has to be free of toxic compounds for distribution in Germany. The charcoal was added (4.6±0.6% by weight) to three treatments, labeled in the experiment as WM with Charcoal (WMC) (Table 1).

We used different sizes of ceramics for the video documentation and the experiment. For the video, eight commercially available ceramics (Ounona 4.5x4 cm Small Mini Terracotta Pot Ceramic, China) were cut in half. The average weight of the ceramics was 15.5±0.6 g and 15.3±0.5 g for the treatment and control for a total sample weight in the containers of 122.3 g and 123.8 g, respectively. For the main experiment, commercially available ceramics (Westerwaei Terrakota Töpfe, Germany; 8 cm inner diameter) were dropped from a height of 80 cm then medium and large pieces were collected and weighed to reach 238±3 g for each treatment (Table 1).

We chose BSF as a model organism, because i) our previous work showed that it is an endogenous species to the Amazon (see S3 Fig), ii) it feeds on a wide variety of organic waste substrates [11, 13, 28], iii) it thrives under tropical climatic conditions [48–51], iv) it is a low-cost waste management strategy [24, 28, 52] and v) it is commercially available in many countries. BSFL were obtained from Hermetia Baruth GmbH, Germany and received in plastic bags two days before the start of the experiment. Prior to shipment the larvae were kept for 24 hours without feeding and were fed with the WM 24 hours before the start of the experiment.

### Experimental set-up

#### Video documentation

Two plastic containers (“reactors”; 11 L *Samla* box 39x28x14 cm IKEA, Germany) were each placed inside a 120 L black plastic bag. The plastic bags kept the reactors dark and collected potential migrating pre-pupae.

The plastic containers were operated as batch reactors, a closed system that has neither inflow of reactants nor outflow of products while the reaction takes place. Before recording we filled each of the reactors with 2.5 kg of the artificial soil substrate, placed 1.5 kg of the WM (enclosed in a rectangle cardboard box without the bottom and top surface, 22x14x6 cm, Rico Design, Germany) on top of the artificial soil, and placed the ceramics on top of the WM.

**Fig 1:**
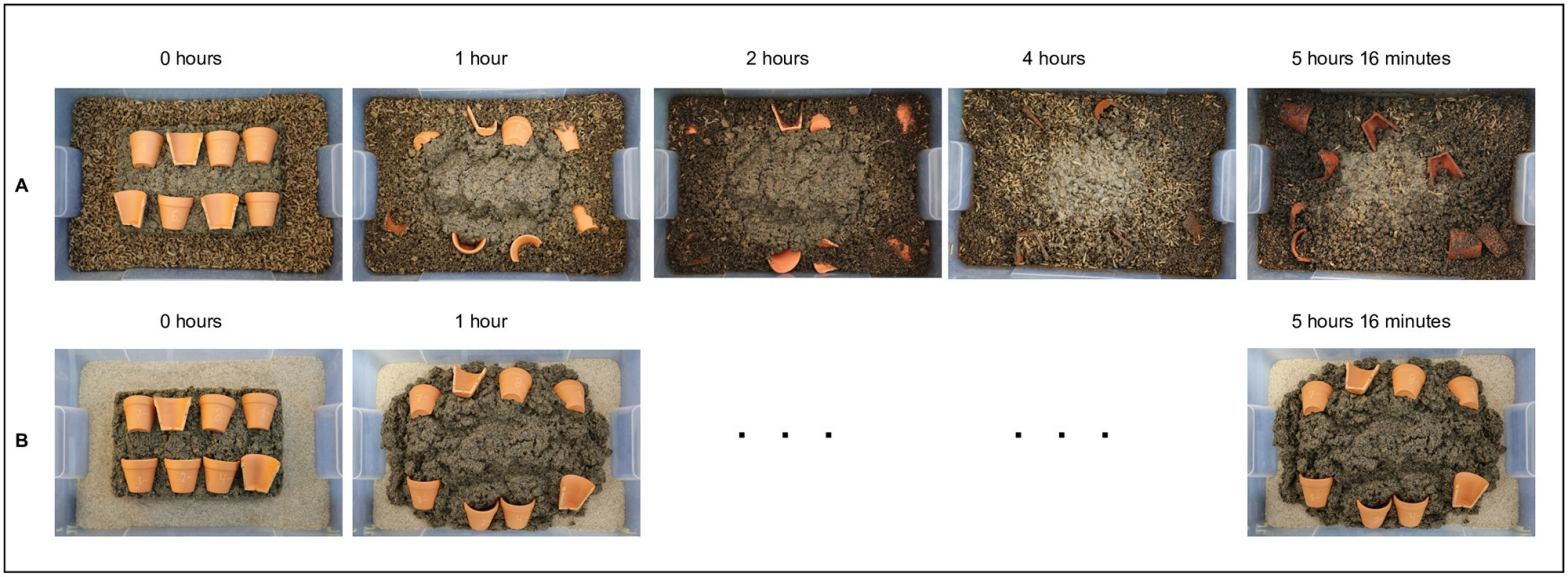
Snapshots of waste turnover and the burial of ceramics by black soldier fly larvae. Larvae of BSF (A) rapidly consume the Waste Model (WM = dog food + pig slurry) while burying ceramic sherds, whereas (B) no sign of bioturbation has been observed in the control treatments.

In the treatment, 600 g of 4th instar larvae [see 53] were added around the cardboard frame containing the waste. The other container served as a control, where no larvae were added. The documentation started when the cardboard frames from the treatment and the control were removed, and finished 5 hours 16 minutes after the removal of the cardboard frame. The video serves as a visual representation of the concept of ceramic-larvae TP formation (Fig 1).

A follow up of the video documentation for a treatment with charcoal was carried out in the same containers using the same 600 g larvae (there was no pre-pupae migration), a second addition of 1,160 g of the WM, 57.5 g of charcoal, and 250.4 g of broken ceramics.

#### Main experiment

Thirty-five reactors (5 L *Samla* box 28x19x14 cm IKEA, Germany) were each placed inside a 120 L black plastic bag. The plastic bags served the same function as in the video. Before the start of the experiment we filled each of the reactors with 2.5 kg of the artificial soil substrate, placed the WM with or without charcoal on top of the soil, and set the ceramics on top of the WM. The experiment started when the larvae (2nd and 3rd instars) were added to the specific treatments (see Table 1).

The reactors were operated as semi-batch flow, allowing reactant addition and/or product removal in time. The WM was prepared 4 hours before addition to the reactors. The larvae were fed during 15 days every 3±1 days. The experiment finished 7 days after the last feeding event to allow migrating pre-pupae to exit the plastic container. Migrating pre-pupae were collected in the black plastic bags. The total duration of the experiment, from first feeding event to sampling, was 22 days.

The reactors were weighed weekly. Samples for physicochemical analysis were collected before the start and at the end of the experiment.

### Methods

#### Incremental soil growth measurement

Incremental growth is defined as the time needed to grow one cm of anthropogenic soil given the constant input of organic matter and nutrients to the artificial soil [16, 17]. For the purpose of this experiment the incremental soil growth rate is the difference of the initial depth to the final depth divided by the time of the experiment:

$$\Delta g=\frac{d_{i}-d_{f}}{t}$$ (1)

where Δg = rate of incremental growth [cm h^-1^ or cm d^-1^], d_i_ = initial depth [cm], d_f_ = final depth [cm], and t = time [h or days]. We consider soil growth or total growth as the expression in the numerator in (1). The final soil depth in the controls and the treatments without larvae was measured as the lowest depth from the top of the container to the surface of the artificial soil or WM, respectively (see S1 Fig). When there was no evident bioturbation, as in the case of the controls, d_i_ and d_f_ were identical and Δg was zero.

The final depth was measured in the corner of the reactors for the video documentation. For the main experiment one final depth measurement was taken for each of the five replicates.

#### Pre-pupae sampling, count, and weight

The BSF pre-pupae are the last motile instar of the immature stage during development. It is a wandering brownish maggot that has lost oral cavities and exits the waste for a drier location where it can remain still and pupate. For commercial purposes BSF are usually collected at this stage [54, 55]. Occasionally BSFL can either accelerate ecdysis and exit the waste earlier, for instance in case of food shortage or unfavorable environment [48, 52, 56], or stay in the substrate when it gets too dry [13].

We searched the plastic bags that the reactors were placed in for the non-feeding, pre-pupal stage, recognizable by its characteristic dark brown color. If the migrating pre-pupae were present in the collecting bags, they were removed, placed in plastic bags, and stored in a freezer at -18°C. We repeated this procedure until the end of the experiment. The pre-pupae were thawed for 24 hours and air-dried for 2 hours before being counted and weighed. Pre-pupae were then dried in an oven (at 105°C) for 24 hours. The average pre-pupae weight (fresh and dry) was calculated by dividing the total weight of all pre-pupae by the number of individuals.

#### Waste model and soil sampling-processing

At the end of the experiment two sets of materials were sampled: the organic layer above the artificial soil, and the sandy layer below. For the organic layer of the treatments, larvae, ceramics, and soil-like material were separated, placed in plastic bags, and stored at -18°C.

Bulk density samples were taken from each of the reactors from the top- and subsoil layers as follows: small PVC rings were used to sample the final material, which was immediately weighed and then left to dry in an oven (at 105°C) for 24 hours, then the dry samples were weighed again.

All soil samples from the two layers were thawed for two hours, thoroughly mixed, and 200 g were freeze dried and stored until further physicochemical analysis. For the amino sugars’ analysis, only samples of the WM + ceramics + larvae and the WM + ceramics were used (n = 10).

### Analysis

#### Soil analysis

Soil pH was determined in 1:2.5 soil/water ratio using a glass electrode. Soil samples from each reactor were sieved to 2 mm, homogenized, and finely ground for combustion analyses. Organic C and total nitrogen (N) contents were determined after dry combustion using a CNS elemental analyzer (vario MICRO cube; Elementar, Hanau, Germany). Total elemental P contents were quantified after hot Aqua regia digestion (mixture of nitric acid and hydrochloric acid), using inductively coupled plasma optical emission spectrometry (ICP–OES; Ultima 2, HORIBA Jobin Yvon, Longjumeau, France).

Phosphorus fractions were determined by a sequential extraction following a modified procedure of Tiessen and Moir [57]. The interpretation of P in each extract is based on an understanding of the action of individual extractants, their sequence, and their relationship to the chemical and biological properties of the soil [58]. Resin-P is reasonably well defined as freely exchangeable and bioavailable inorganic P (Pi). The bicarbonate-extract (0.5 M NaHCO_3_) presumably removes labile Pi and organic P (Po) sorbed on soil minerals and a small amount of microbial P. The hydroxide-P (0.1 M NaOH) would represent Al- and Fe-associated Pi and Po that are strongly held to soil surfaces by chemisorption. The dilute acid-Pi (1M HCl) is clearly defined as Ca-bound Pi, and there is rarely any Po in this extract.

For all samples, 0.5 g of soil was weighed (in duplicates) into a 50 mL centrifuge tube, then 30 mL of deionized water and two strips of anion-exchange resin (1x6 cm; Ionac MA-7500, Sybron Chemicals, Birmingham, NJ, USA) were added to the tubes. The tubes were shaken for 16 h on a horizontal shaker. After the resin strips were removed, they were placed in a clean 50 mL tube with 20 mL of 0.5 M HCl, and shaken for 16 h. Tubes with the soil residues were centrifuged for 20 min at 0°C at 25,000xg, and water was discarded. Then, 30 mL of 0.5 M NaHCO_3_ (pH 8.5) was added to each tube, vortexed, shaken for 16 h, and centrifuged as above. The supernatant was filtered through a filter paper (Quantitative Filter Paper ashless, DP 5892125, Hahnemuehle, Dassel, Germany). The extraction process was repeated using 30 mL of 0.1 M NaOH and 1 M HCl as above. For each extract, P_i_ concentrations were determined by molybdenum-blue method [59] using a spectrophotometer (SPECORD 205, Analytik Jena, Jena, Germany). Pt concentrations in the extracts were quantified by ICP–OES (Ultima 2, HORIBA Jobin Yvon, Longjumeau, France). P_o_ concentrations were calculated as the difference of P_t_ and P_i_ in each extract.

The amino sugar analysis was conducted according to the protocol of Zhang et al. [60]. In brief, amino sugars were hydrolyzed from 150 to 200 mg soil, corresponding to 0.3 mg N per sample, using 10 mL of 6 M HCl for 8 h at 105°C. After filtration, rotary evaporation, and redissolving with Millipore water, samples were adjusted to pH 6.6–6.8 and centrifuged (1,500xg for 10 min). The clear supernatant was freeze-dried, washed three times with 1.5 mL methanol, transferred into reactivials, evaporated with a stream of pure nitrogen (> 99.9 n/n%) and finally derivatized to aldononitriles as described in the original methodology. Final amino sugar quantification was done by gas chromatography-mass spectrometry (GC–MS 6890N, Agilent Technology, California, USA). Myo-inositol was added as first internal standard to the hydrolysate and ß-endosulfan was used to assess the recovery of myo-inositol. It averaged only 52% for all samples, thus pointing to the possibility of some amino sugar losses during sample processing. Nevertheless, we did not observe any correlation between internal standard recovery and amino sugar concentrations or patterns, suggesting that if some losses occurred, they were likely nonspecific, not affecting their potential use for tracking chitin residues in the samples.

#### Data analysis

All data were analyzed using Stata/SE 14.2 for Mac (64-bit Intel) and plotted using R [61]. Independent group t-tests were used to compare the soil growth between treatments. After checking for heteroscedasticity using the Breusch-Pagan test, BSFL treatments were compared to the treatments without larvae for each of the different waste types. A t-test was used to compare the amino sugars present in the WM + ceramics + larvae to the concentration in the WM + ceramics but without larvae. A t-test also assessed the effect on soil growth from the different substrate types. After testing for normal distribution using the Shapiro-Wilk test, physicochemical parameters of the final material were compared to those of the original WM using a single sample t-test: if the corresponding two-tailed p-value was less than 0.05 it was concluded that the final material was significantly different from the original material. The reduction percentage for the different physicochemical concentrations was calculated as:

$$Conc_{red}=(1-\frac{Residue_{Conc}}{Waste_{Conc}})\times 100$$ (2)

where Residue_Conc_ and Waste_Conc_ were the total concentration of elements in the treatment residue and the original WM, respectively. We assessed the statistical reliability of the relationship between different initial materials and the different treatments with linear regressions using charcoal and presence or absence of larvae and/or ceramics to predict specific changes on physicochemical soil parameters. All data of the regression model were tested for normality and homoscedasticity of residuals. Influential values were predicted and cut-off using Cook’s D overall measure of influence.

## Results

### Video documentation

The video gives evidence of the high waste to soil conversion and bioturbation potential of the BSFL. The larvae consumed all the waste at a high rate within a few hours and displaced and buried the ceramics, accounting for high bioturbation. Ceramics were brought back to the surface, buried, and brought back again by the larvae’s action while feeding (see Fig 1). Soil growth in the treatment with larvae was 2.86±0.19 cm at the end of the 5 hours and 16 minutes of the experiment, resulting in an incremental growth rate of approximately 0.5 cm per hour. The control without larvae showed no hints of decay, no buried ceramics, or evident bioturbation, as shown in Fig 1 and S2 Fig. We thus conclude that BSFL can displace even large, broken ceramic sherds and consume organic waste mixed with charcoal, as shown in the follow-up video (see S1 Video at https://osf.io/x35kn/).

After the first feeding event with the WM, BSFL remained inactive under the faunalmantle without feeding for five days. The larvae started feeding again when more organic waste (WMC) and ceramics were placed on the top surface (see S1 Video at https://osf.io/x35kn/).

### Main experiment

#### Soil growth

**Fig 2:**
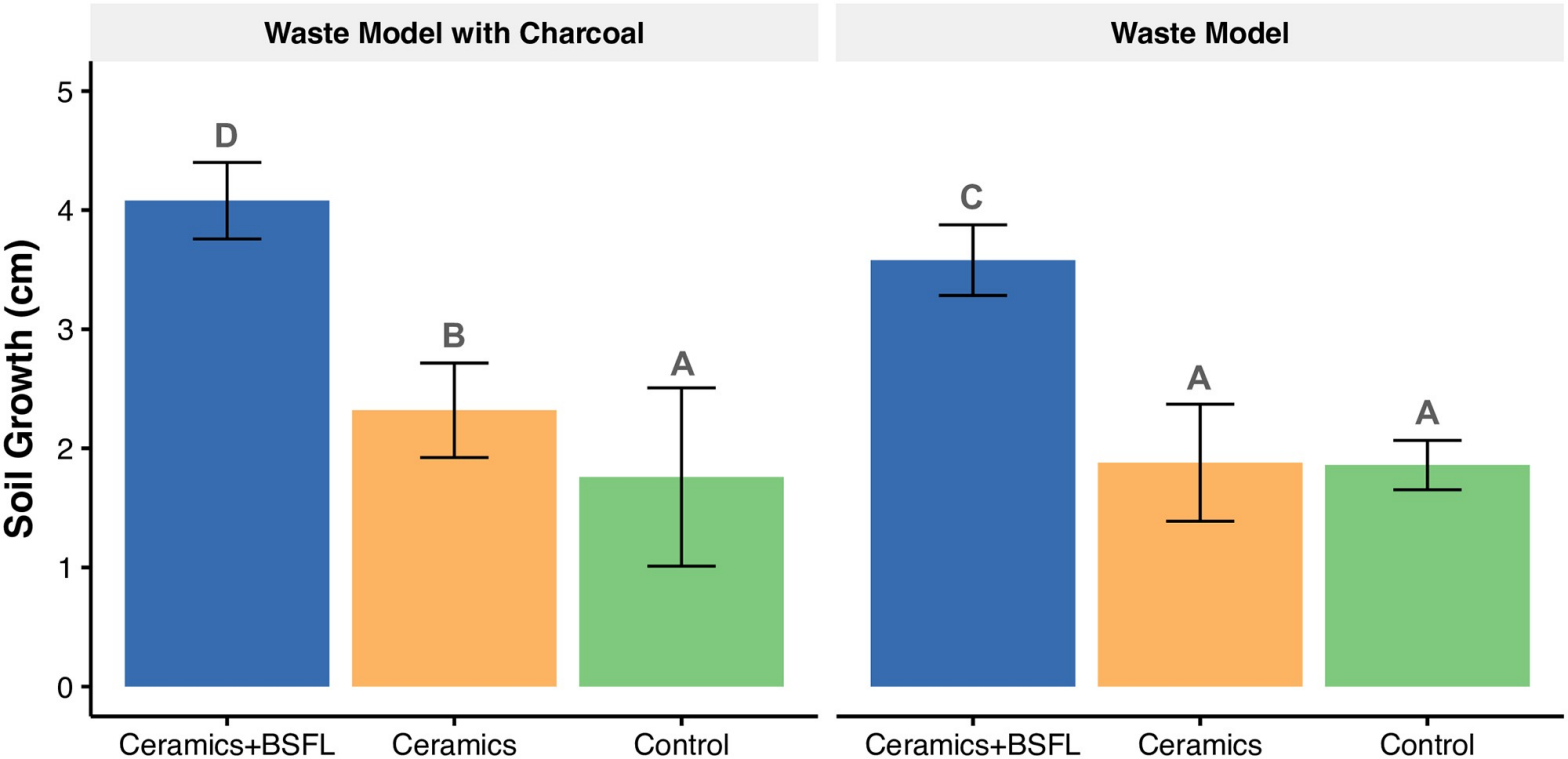
Black soldier fly larvae (BSFL) increase soil growth in comparison to treatments without larvae. Larvae fed with the Waste Model with charcoal (WMC) presented the highest soil growth among all the treatments and waste model types. Treatments with ceramics in the absence of larvae and absolute controls showed no significant differences. Five replicates per treatment. The error bars represent the 95% confidence intervals (CI) of independent group t-tests. Treatments sharing a letter are not significantly different at the 5% level.

Soil growth was higher in all the BSFL reactors when compared with the treatments without larvae. The difference of means in soil growth between BSFL treatments and the other treatments without larvae is different from 0 (p<0.05) (Fig 2). When the charcoal was present, the treatment with larvae and ceramics fed with WMC (Δg = 0.18±0.014 cm day^-1^) showed significantly higher growth (p = 0.013) than the treatment fed with the standard WM without charcoal (Δg = 0.16±0.014 cm day^-1^). The highest incremental growth rate was lower than in the video and reached 1.8±0.1 mm day^-1^.

Although the main experiment lasted about 22 days longer than the video documented experiment, similar bioturbation patterns were observed in the BSFL treatment as opposed to the treatments without larvae: BSFL rapidly consumed the WMs, mixed their soil-like material with the artificial soil, thus creating an even topsoil surface (faunalmantle), and buried the ceramic sherds (S2 Fig). In general, a more even soil layer was observed in the treatments with larvae than in those without larvae.

#### Physicochemical soil properties

**Fig 3:**
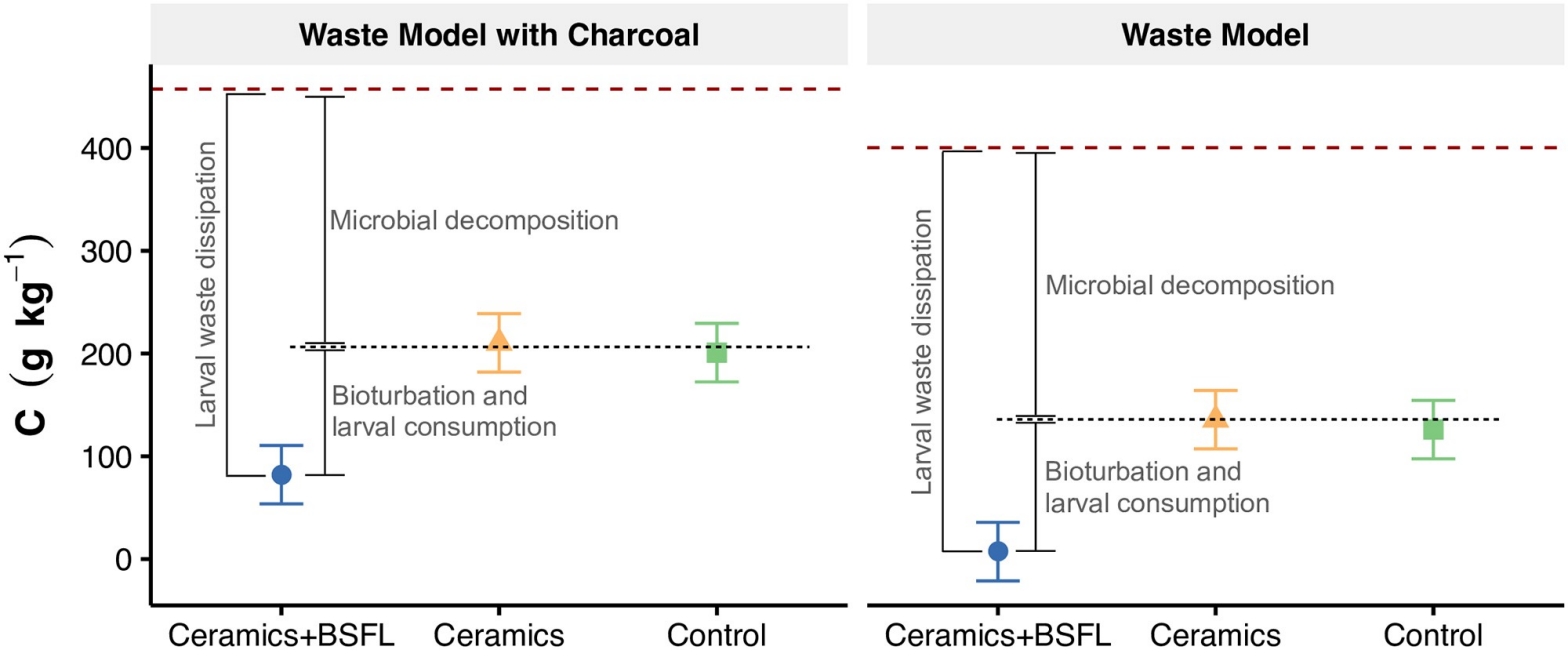
Final total carbon concentrations as influenced by the waste model and treatments. The presence of larvae significantly decreased soil C content relative to the other treatments. Herein, the waste model with charcoal (WMC; 5% charcoal w/w) exhibited higher C concentration than the waste model without charcoal (WM). The error bars represent the 95% confidence intervals (CI) and the red-dashed lines represent the total organic C concentration in the initial waste material.

The total organic carbon (TOC) concentrations in the organic waste exceeded 40% (Fig 3; red dotted line). After the experiment was stopped, TOC concentration had decreased significantly (p<0.001) in the organic waste layer of all treatments. The WM + ceramics + larvae treatment exhibited the highest reduction in TOC stocks (94.4±0.8%), followed by the WMC + ceramics + larvae treatment (85.4±2.9%). This additional decline in TOC concentration in the BSFL treatment went along with a deeper mixing of the organic material within the mineral soil (S1 Fig and S1 Table) and consequent high soil growth (Fig 2).

The presence of charcoal in the WMC treatment resulted in a higher C concentration than in the respective WM treatment (Fig 3, left). This effect was continuous until the end of the experiment (p<0.001). Again, the presence of BSFL significantly reduced TOC contents, whereas the effect of the ceramics on the TOC losses was not significant (p = 0.62; Fig 3, right).

The pH in the final decomposed waste material had increased (p<0.05) relative to the initial material, except for the WM treatment, which did not change significantly over time (Table 2). There was also an increase in pH in the BSFL treatments compared with the treatments without larvae.

**Table 2:** Physicochemical parameters of the organic waste materials at the beginning and after 22 days after adding black soldier fly larvae.

Soil Parameter	Initial Material*		Final Material after 22 Days*					
	WMC (95%CI)	WM (95%CI)	WMC (95%CI)	WM (95%CI)	WMC+CS (95%CI)	WM+CS (95%CI)	WMC+CS+L (95%CI)	WM+CS+L (95%CI)
**pH**	7.13 (6.97–7.29)^B^	6.80 (6.65–6.97)^A^	7.18 (7.02–7.34)^B^	6.85 (6.69–7.01)^A^	7.58 (7.42–7.74)^C^	7.25 (7.09–7.41)^B^	7.98 (7.82–8.14)^D^	7.65 (7.49–7.81)^C^
**N**	45.49 (43.0–48.0)^C^	44.7 (42.15–47.21)^C^	15.42 (13.0-.17.95)^B^	14.61 (12.08–17.14)^B^	16.68 (14.15–19.21)^B^	15.9 (13.34–18.40)^B^	4.44 (1.91–6.98)^A^	3.63 (1.1–6.16)^A^
**C/N**	12.01 (11.03–13.0)^B^	7.38 (6.41–8.36)^A^	12.95 (12.0–13.93)^B^	8.33 (7.35–9.30)^A^	12.84 (11.87–13.82)^B^	8.22 (7.24–9.19)^A^	13.0 (12.02–14.0)^B^	8.37 (7.39–9.35)^A^
**∑ Pi**	4669 (4135–5203)^EF^	4960 (4426–5494)^F^	3196 (2662–3730)^BC^	3487 (2953–4021)^CD^	3767 (3233–4301)^CD^	4058 (3525–4592)^DE^	2095 (1561–2629)^A^	2386 (1852–2920)^AB^
**∑ Po**	4500 (3955–5046)^D^	4793 (4247–5338)^D^	1684 (1139–2230)^BC^	1977 (1431–2522)^C^	1817 (1272–2363)^BC^	2110 (1565–2656)^C^	855 (310–1401)^A^	1148 (603–1694)^AB^

WM = waste model, WMC = waste model with charcoal, CS = ceramic sherds, L = larvae. Concentrations of N and P are given in g kg^-1^ soil, those of amino sugars are expressed in g kg^-1^ N.

*Treatments sharing a letter after the 95%CI are not significantly different at the 5% level for the respective soil parameter.

The concentrations of N followed that of TOC in the course of the experiment (Table 2 and S1 Table). N content’s most relevant decline occurred in the larval treatments from around 45.5 mg g^-1^ to almost 4.4 mg g^-1^ for the WMC + larvae and from 44.7 mg g^-1^ to 3.6 mg g^-1^ for the WM + larvae treatment. This is typical for mixing organic materials with mineral compounds and results in a dilution of element concentrations by siliceous matter. Nevertheless, these declines in N concentrations were did not affect C/N ratios in all treatments during waste decomposition. The charcoal addition resulted in higher C/N ratios than in the other WM treatments (X^2^ = 88.52, p<0.0001).

The proportion of N in the form of amino sugars in total soil and waste N did not differ significantly among trials (p>0.05), averaging 97 ± 30 g kg^-1^ N in the trials with larvae and 94 ± 19 g kg^-1^ N in the trials without. Also, glucosamine contents did not differ significantly among trials. The contributions of glucosamine to total N even tended to be lower in the trials with BSFL (45 ± 17 g kg^-1^ N) than in those without (58 ± 13 g kg^-1^ N; p > 0.05), thus not pointing to any mechanism of glucosamine enrichment with BSFL.

Apart from N, an essential nutrient for plants and microbes from a quantitative point of view is P. Similar to N, the sum of the inorganic (∑Pi) and the organic contents (∑Po) for the different P fractions showed the most drastic reduction in concentration from the original material to the residue of the larval treatments (Table 2). The most remarkable P fractions’ concentration decrease occurred in the treatments with larvae: concentrations dropped by a factor of about 5 for ∑Po and by a factor of 1.8 for ∑Pi from the WM original material and by a factor around 4 for ∑Po and 2.6 for ∑Pi from the original WMC material (Table 2). These concentration changes were evident in all fractions. Unfortunately, a reliable calculation of P stocks failed due to larvae’s presence distorting a reproducible bulk density assessment.

**Fig 4:**
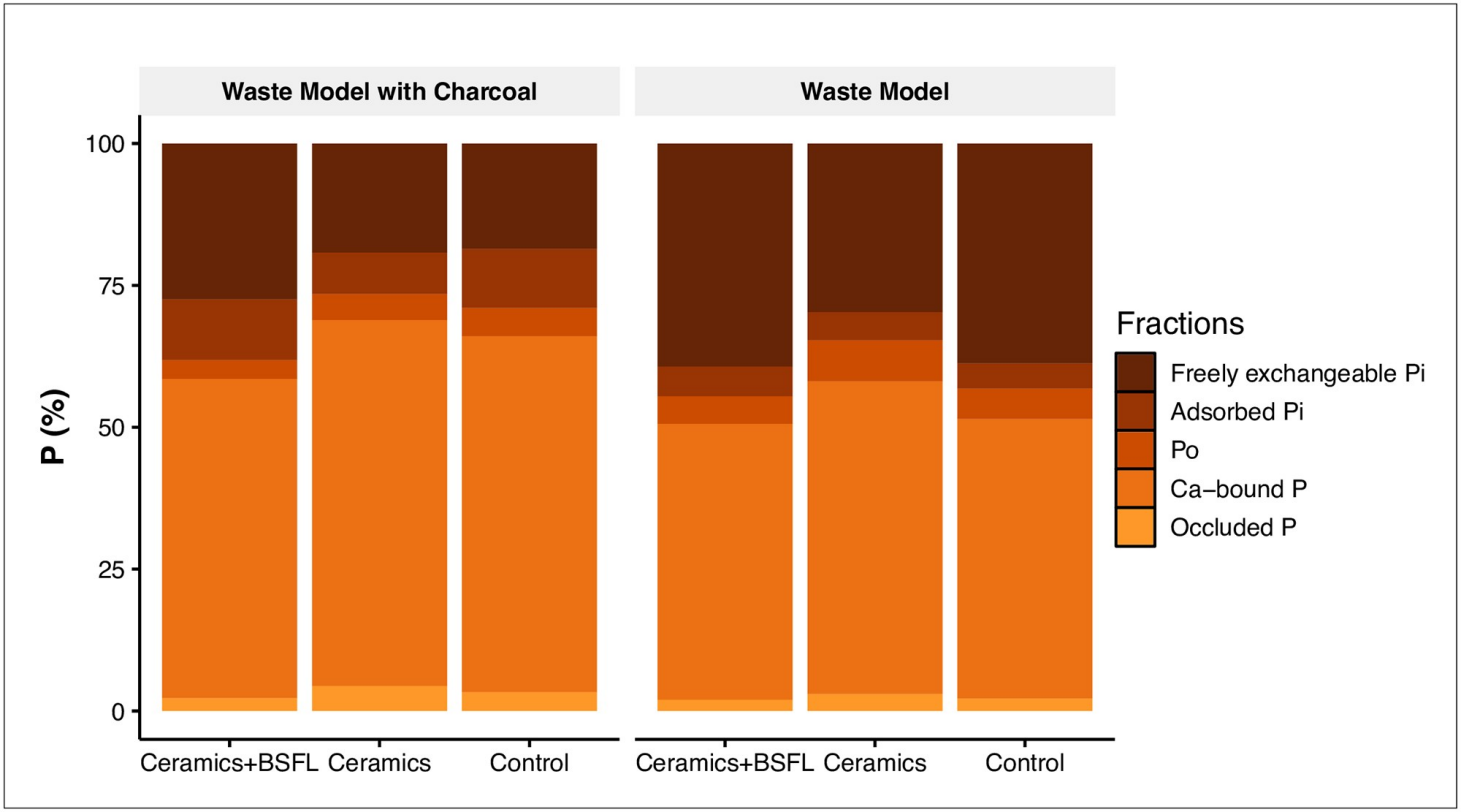
Relative distribution of the P fractions in the treatments with and without black soldier fly larvae (BSFL), ceramics, and for the waste models with (left) and without charcoal (right). Freely exchangeable inorganic P (Pi) = resin-Pi, adsorbed Pi = bicarbonate (0.5 M NaHCO3)-and sodium hydroxide (0.5 M NaOH)-extractable Pi, organic P (Po) = bicarbonate Po + hydroxide-Po, calcium-bound P = dilute acid-extractable (1 M HCl)-P, and occluded + residual P = concentrated-acid (97% HCl) extractable P and acid-digestible P (1:3 concentrated HNO_3_: concentrated HCl mixture).

We also calculated the relative proportions of total P within each of these fractions to understand changes in the P distribution pattern among different chemical fractions. The result showed that this distribution in the WMC treatments differed from those in the WM treatments (Fig 4). Most P was found as HCl-extractable P, which typically isolates Ca-bound P. Respective pool sizes ranged from 49% to 55% of total P in the WM and from 56% to 64% of total P in the WMC treatments. Freely exchangeable P was the second most abundant form, ranging from 30% to 39% of total P in the WM treatments and from 19% to 27% of the WMC treatments. The presence of larvae increased the percentage of the freely exchangeable Pi pool relative to the other treatments.

#### Pre-pupae migration and weight

**Fig 5:**
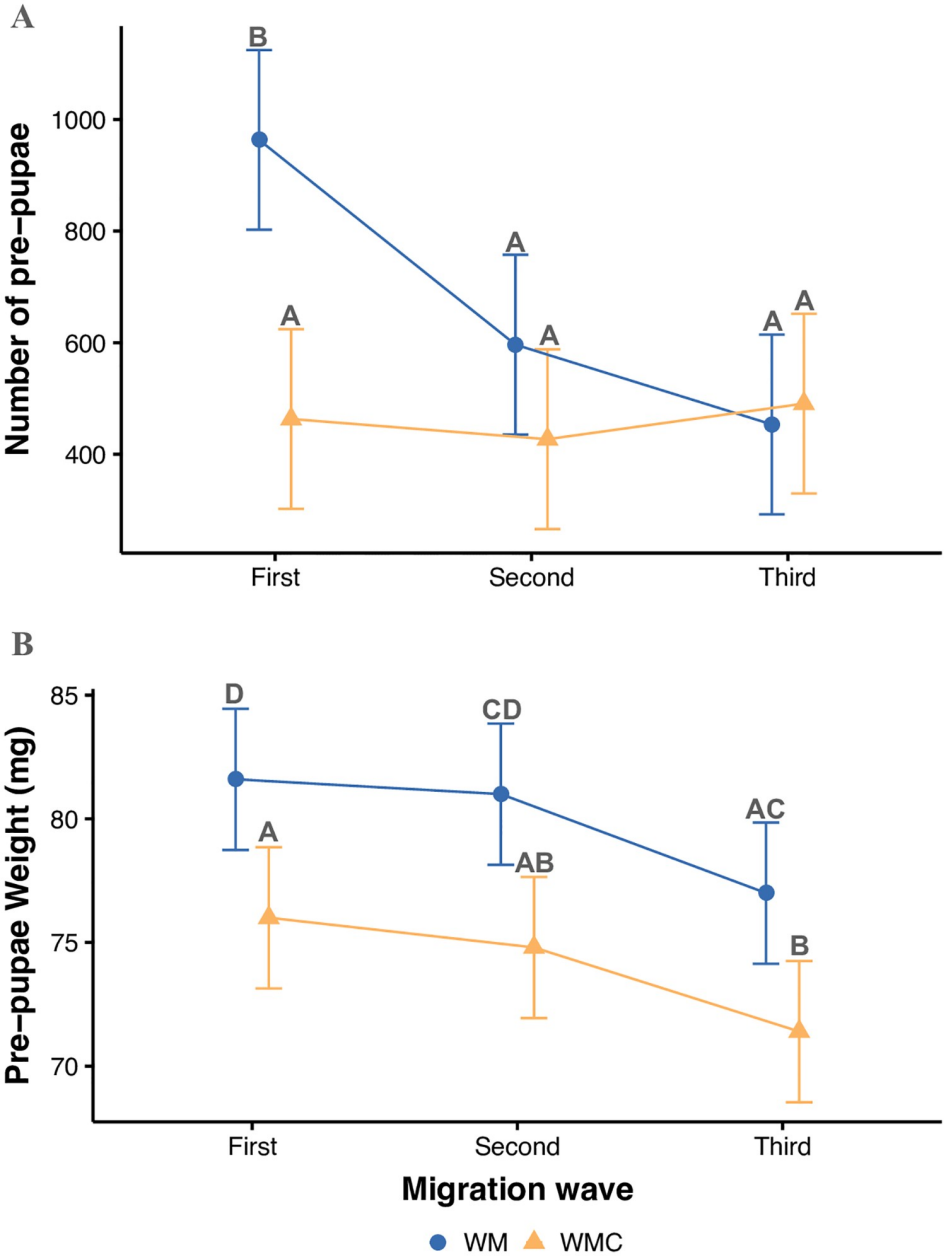
Charcoal delays development and has a negative effect on black soldier fly larvae (BSFL) pre-pupal weight. Charcoal (A) keeps the migration of pre-pupae constant during the duration of the experiment, whereas the waste model (WM) triggers a rapid development evident in the first migration wave that further decreases in the second and third migration waves. The WM-fed pre-pupae weight is (B) higher in all migration waves when compared to pre-pupae fed with the waste model that contains charcoal (WMC). The error bars represent the 95% confidence intervals (CI). Treatments sharing a letter are not significantly different at the 5% level.

Three migration waves occurred during the main experiment. The first had a significantly higher number of pre-pupae exiting from the WM reactors than from the WMC reactors (X^2^ = 10.89, p < 0.01). The presence of charcoal delayed the development of the BSFL (Fig 5A). Further statistical analysis of the individual pre-pupal weight confirmed that charcoal negatively affected the dry weight of BSF pre-pupae (Fig 5B). Pre-pupae weight was significantly higher for insects fed with WM (80±4 mg larva^-1^ fresh weight) than for those fed with WMC (74±3 mg larva^-1^ fresh weight) (X^2^ = 25.37, p<0.0001). A weight reduction tendency was observed in the third migration wave, probably caused by the scarcity of food when we stopped feeding the larvae seven days before the end of the experiment.

## Discussion

There is no comparative study that links ceramics, fly larvae, and soil formation to the best of our knowledge. Most studies focus on BSF and other fly species relate to food and feed production [52, 62], waste management [11, 28], and/or production of cleaner residues [11, 24], whereas TP studies focus on the origins of these Amazonian soils [7, 63] or the application of the so-called “key components” of TP, like charcoal [64, 65]. In turn, our results make it likely that such a connection exists: the BSFL rapidly decomposed waste and mixed it into the soil, even with larger ceramic fragments as commonly observed in TP sites. Our WM encompasses general archaeological evidence in that it included the presence of ceramics and charcoal and feces, though in our case, not from humans but from pigs.

### Video documentation: Bioturbation by BSFL

Armour-Chelu and Andrews [66] pinpoint that “bioturbation of soils by invertebrates is difficult to infer directly as most of these animals are soft bodied so that their remains are only rarely recovered from paleosols” (p. 440). BSFL have cuticles that are not entirely soft, yet most of the larvae exit the waste once they have achieved certain development, thus decreasing the chances of finding them in a long-term archaeological context. The video documentation positions the BSFL as relevant bioturbation organisms with soil growth rates accounting for 0.18 mm day^-1^ (main experiment) to maximum rates of 0.5 cm h^-1^ (video documentation).

From the short life cycle of BSF (about 45 days), only about 18 days are spent as larvae. This period represents the time BSFL actively drive waste decomposition and directly interact with the topsoil layer. They depend on the availability of waste input that is not typically considered in soil surveys. Therefore, BSF are not frequently encountered in soil studies because they only inhabit the middens during the initial stages of waste decomposition. Our study’s high bioturbation rates are not likely constant throughout the year but plateau once the waste is decomposed and the larvae have emerged the site as adult flies.

A single BSF female can populate large areas given that waste availability and optimal conditions are met. One BSF female can lay between 500 to 1,200 eggs that hatch in approximately four days, depending on environmental conditions. The Amazon basin offers perfect climatic conditions for these organisms to thrive, similar to our greenhouse experiment conditions. The missing component would be the waste generated by human activities. However, all archaeological evidence suggests stable pre-Columbian societies in the Amazon. They settled for prolonged periods in sedentary communities extending from the north-western area of the Amazon basin [67, 68], Santarem [69], throughout central Amazonia [70, 71], and along the entire southern rim of the Amazon basin [72]. Estimations of pre-Columbian population numbers often increase with new data unearthed in the Amazon. In recent years, a population of 15 million people has been estimated [73–75]. Although still highly speculative, large pre-Columbian populations would have had higher waste production, ensuring a stable substrate source for BSF colonies to develop. We, therefore, suggest that our study points to a causal interaction between past TP formation and the presence of BSF.

### Main experiment

#### Soil growth

The main experiment confirmed that the larvae buried larger ceramic sherds for the two types of waste. Nevertheless, no apparent horizontal movement of the ceramic sherds was observed, and soil growth was much slower than observed in the video documentation. We assume that the smaller area of the containers, compared to the first experiment and the larger ceramic sherds, caused the ceramics to remain static. BSFL’s ability to move more massive ceramic pieces should now be tested in a larger experimental setting, ideally under field conditions.

For both WMs, all treatments with BSFL increased soil growth and formed a faunalmantle compared to the treatments without larvae. These results confirm that in the short term, during the transition from waste decomposition to nutrient incorporation into the soil, BSFL promote soil growth and accelerate incorporation of waste debris into the soil compared to microorganisms’ activity. Assuming that our data could be transferred to tropical field conditions, our results suggest that the estimation of Smith [16] of TP incremental growth of 1 cm per every ten years might be an underestimation. Instead, as Neves et al. [17] proposed, TP formation might be strongly dependent on intense human activity, which translates to constant disposal of ceramic sherds and organic waste and therewith potential for BSFL colonization.

It is essential to recognize that although the final depth measurements for the second experiment were taken seven days after the last feeding event to allow migrating pupae to leave the reactors, a high number of larvae still remained under the topsoil and contributed to a higher growth accounting for the volume displaced by the BSFL. Therefore, a considerable fraction of this soil growth is larval biomass. Larvae feed on the organic waste model, grow and create an even layer of humus-like material or faunalmantle. The phenomenon that some larvae stayed in the reactor was observed in other studies with BSFL and attributed to the reactors’ dry conditions [12]. Consequently, soil growth in the presence of BSFL under field conditions will likely depend on moisture levels and will probably be high in moist conditions prevalent throughout most of the Amazon basin.

#### Alterations of soil properties

All physicochemical parameters analyzed in the second experiment changed from the original waste material to the final larval residue. The general increase in pH values confirms earlier observations by Lalander et al. [12], who reported an increase of the pH from 5.6 in the inflow material to 8.1 in the outflow material of BSFL waste-recycling systems. Liu et al. [24] assigned similar increases in pH to substrate alkalization by ammonia formation. In our experiment, the highest pH values were recorded when larvae were present, thus affirming their role in decomposing organic matter and subsequent ammonia formation. The higher pH in the final material also reflects the incorporation of waste into the alkaline artificial soil.

Due to the mineralization of organic carbon to CO_2_, TOC concentrations usually decrease during waste decomposition. Yeton et al. [76] reported mineralization rates between 54% and 69% of total added C after 75 days of decay using a combined treatment with common housefly and BSF. Our results suggest similar and even higher mineralization of TOC (Fig 3), most likely because our waste contained less lignocellulosic material than the material used by Yeton and colleagues, who also placed the material in meshed litter bags in an Acrisol (15 cm depth) and not on the top of the soil as we did. Moreover, our experimental design allowed BSF to mix the residue with the artificial soil, causing a further dilution effect in TOC concentrations with increasing soil growth in addition to the TOC loss by mineralization (Figs 2 and 3). However, Liu et al. [24] outlined that the strong respiratory action of microorganisms inside the insects’ intestines contributed to accelerated waste degradation.

A BSFL treatment can be considered a well-ventilated system due to intense bioturbation and movement by the larvae during waste processing, as evidenced in our video. With temperatures above 25°C and high pH values as observed, the volatilization of ammonia is enhanced [12], and may, thus, explain losses of N in the BSFL treatments. Similar to TOC, mixing with the artificial soil contributed to the decline in N concentrations. A small amount of N may have left the treatments with the migrating larvae. The lack of significant glucosamine accumulation implies that few if any molted cuticles of BSF [41] contributed to the systems’ amino sugar patterns.

As opposed to C and N, P-bearing moieties are not mineralized into volatile compounds during organic matter decomposition. Also, the leaching of P out of the system was not possible in our reactors. An increase in P concentration during waste decomposition and P accumulation in the final product might be expected. However, early studies showed that BSFL could remove approximately two third of the P present in the dry matter of cow manure [77] and almost half of the P in pig manure [78]. Contrary to that, Lalander et al. [12] and Liu et al. [24] reported an increase in total P concentration for all treatments. Our results support the earlier findings that BSF larvae remove excess P from organic waste materials, likely leaving the waste boxes as part of the pre-pupae biomass, diluting the P while mixing with the sandy substrate, or both.

Ca-P and freely exchangeable Pi were the most relevant pools contributing to the total P stocks in the final material (Fig 4). Noticeably, the proportion of available Pi was lower in WMC than in WM treatments, whereas the proportion of Ca-P was higher in WMC than in WM treatments. This supports the assumption that charcoal contributes to stabilizing both organic matter [79–81] and associated nutrients. The pronounced effects on total P concentration and P fractions in the WM or WMC + ceramics + BSFL treatments compared to the WM or WMC + ceramics treatments suggest that the ceramic sherds did not act as a P source and that the effect of the BSFL was more relevant for the supply of available P than the effect of the ceramics only.

In principle, the BSFL may affect the soil directly or via its associated microbial activity. In this study, microbial activity was not measured, but we measured microbial necromass formation via amino sugar analyses. We did not find evidence of larvae having a distinctive effect on the soil amino-sugar concentrations. Likely, because our experiment’s duration was too short to significantly affect the pool of soil microbial residues with expected turnover rates of a few years [39] because the larvae’s pronounced effect on waste-degrading microorganisms did not exist, or both.

### Pre-pupae migration and weight

The time elapsed until the first observation of pre-pupae was 13 days from the beginning of the experiment, similar to reported times in literature [82]. This duration is a very broad approximation because the time when the first larvae develop into the pre-pupae stage depends on the initial instar at the beginning of the experiment. Nevertheless, considering that humidity and temperature were kept constant for all the reactors, the significantly greater number of pre-pupae in the first migration wave of the WM reactors suggests that larvae completed their development faster in the reactors fed with WM than those fed with WMC (Fig 5A).

The pre-pupae in our substrates were very small (<90 mg larva^-1^). Lalander et al. [82] reported that the pre-pupae weighed >210 mg larva^-1^ for most of their waste substrates but also found a low weight for larvae fed with digested sludge (70 mg larva^-1^). Larvae transport, substrate change, and new conditions might have caused stress and a subsequent low weight in our pre-pupae. Furthermore, larval weight might have negatively been affected by the addition of charcoal, as indicated by the delayed development in the WMC treatments compared to the WM ones. The stabilizing effect of charcoal on SOM may have led to a shortage or lack of essential nutrients needed to support the growth of the insects [82, 83]. Nevertheless, the BSFL survived these conditions and are less likely affected by charcoal’s toxicity than earthworms, thus rendering BSFL as a more likely candidate for intensive bioturbation in waste middens in tropical environments compared to earthworms or termites.

## Conclusion

The batch reactors with larvae fed on a waste and soil model demonstrate the potential of BSFL for waste decomposition and conversion into a fertile soil-like material. With the amounts of waste applied in this study, these processes occurred within hours, indicating a much faster soil development rate than assumed in traditional bioturbation models and related soil or TP formation. BSFL bioturbation includes the burying of ceramic sherds and represents a new paradigm that could explain how potsherds were transported into deeper layers in Amazonian Anthrosols profiles. Considering the archeological evidence of the extent of pre-Columbian civilizations and the type and amount of waste available from these settlements, we suggest that waste decomposition by BSFL may have contributed to the development of TP soil profiles. However, as our study was limited to a short-term greenhouse experiment, the effects of BFSL on soil formation processes in tropical conditions now need to be corroborated in longer-term field studies in an Amazonian setting. They could be further extended by more detailed assessments of the potential for waste processing based on BSF reproduction cycles and for remediation of toxic waste components.

## Supporting information

S1 Fig First experiment’s upper and side views of the treatment and control. (**A**) Upper/Frontal view of the treatment with larvae (left) and the control (right). The lateral view of the (**B**) treatment evidences how fast (5 hours 16 minutes) BSF larvae can process organic material and displace ceramics laterally and vertically and incorporate the processed material within the topsoil creating a faunal mantle. By comparison (**C**) the control without larvae after the same period shows no relevant activity. The incremental soil growth is the difference between the initial depth (d_i_) of the soil model and the final depth (d_f_) at the end of the experiment. The control shows no incremental growth as there was no evident bioturbation of the soil model.
(DOCX)

S2 Fig BSFL’s ceramics burial and topsoil layer (faunal mantle) formation. A pretty even soil layer is observed in the treatments with larvae whereas treatments that have only ceramics or waste remain at initial stages of waste decomposition.
(DOCX)

S3 Fig Adult Black Soldier Fly (BSF) in the Amazon. BSF can be found distributed below altitudes of 2000 m (most of the Amazon basin). Photo: Juan M. Orozco-Ortiz. Leticia, Colombia.
(DOCX)

S1 Table Carbon (C) and Nitrogen (N) concentrations by layer. (DOCX)
