# Supplementary material for: Bioturbation by black soldier fly larvae—Rapid soil formation with burial of ceramic artifacts
Source: PLoS One. 2021 Jun 2;16(6):e0252032. doi: 10.1371/journal.pone.0252032 (PMC8171933; doi:10.1371/journal.pone.0252032)
Supplement: S1 Fig — (A) Upper/Frontal view of the treatment with larvae (left) and the control (right). The lateral view of the (B) treatment evidences how fast (5 hours 16 minutes) BSF larvae can process organic material and displace ceramics laterally and vertically and incorporate the processed material within the topsoil creating a faunal mantle. By comparison (C) the control without larvae after the same period shows no relevant activity. The incremental soil growth is the difference between the initial depth (di) of the soil model and the final depth (df) at the end of the experiment. The control shows no incremental growth as there was no evident bioturbation of the soil model. (DOCX) [file pone.0252032.s001.docx]

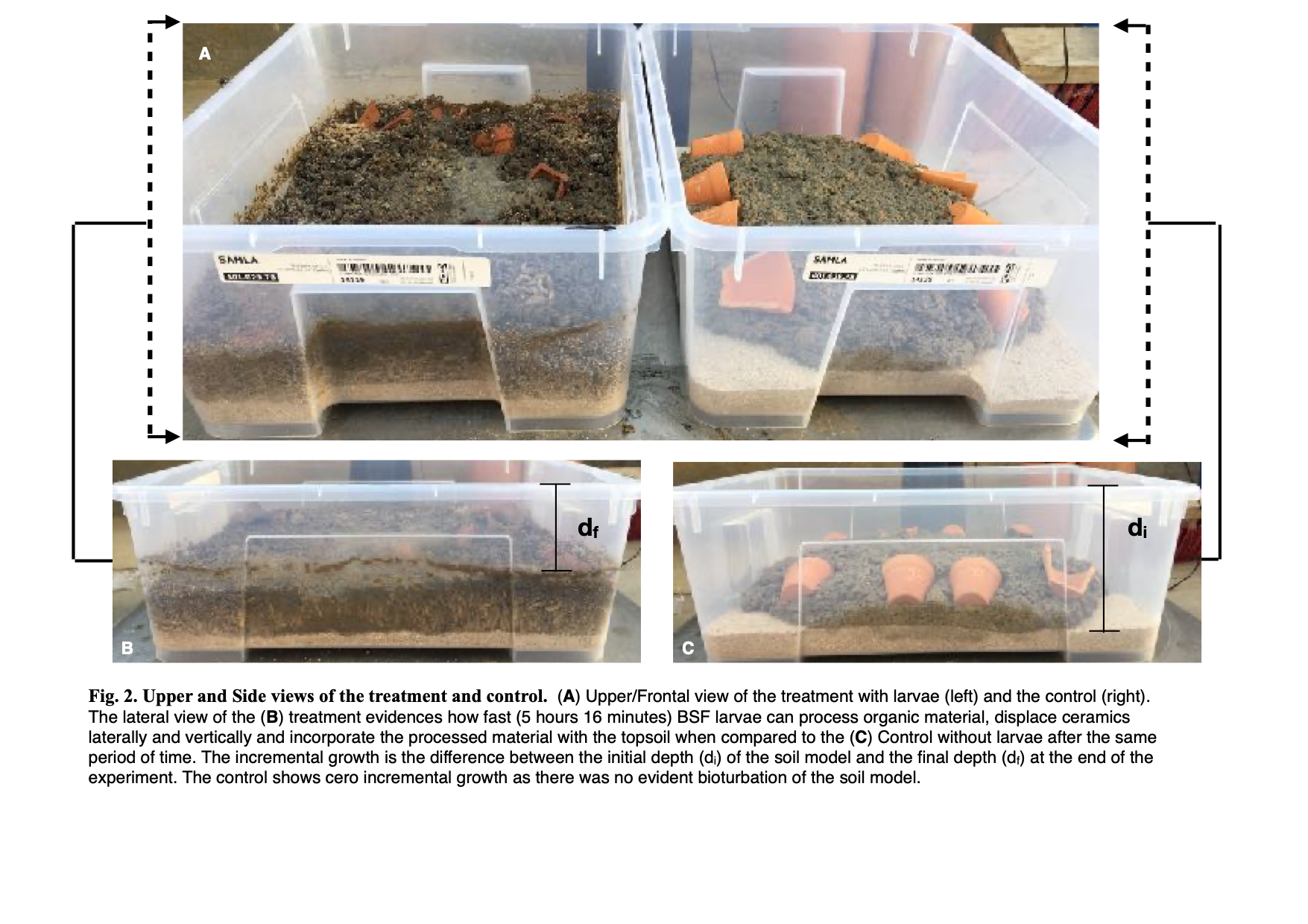


S1 Fig. First experiment’s upper and side views of the treatment and control

(**A**) Upper/Frontal view of the treatment with larvae (left) and the control (right). The lateral view of the (**B**) treatment evidences how fast (5 hours 16 minutes) BSF larvae can process organic material and displace ceramics laterally and vertically and incorporate the processed material within the topsoil creating a faunal mantle. By comparison (**C**) the control without larvae after the same period shows no relevant activity. The incremental soil growth is the difference between the initial depth (d_i_) of the soil model and the final depth (d_f_) at the end of the experiment. The control shows no incremental growth as there was no evident bioturbation of the soil model.
