# Supplementary material for: Bioturbation by black soldier fly larvae—Rapid soil formation with burial of ceramic artifacts
Source: PLoS One. 2021 Jun 2;16(6):e0252032. doi: 10.1371/journal.pone.0252032 (PMC8171933; doi:10.1371/journal.pone.0252032)
Supplement: S2 Fig — A pretty even soil layer is observed in the treatments with larvae whereas treatments that have only ceramics or waste remain at initial stages of waste decomposition. (DOCX) [file pone.0252032.s002.docx]

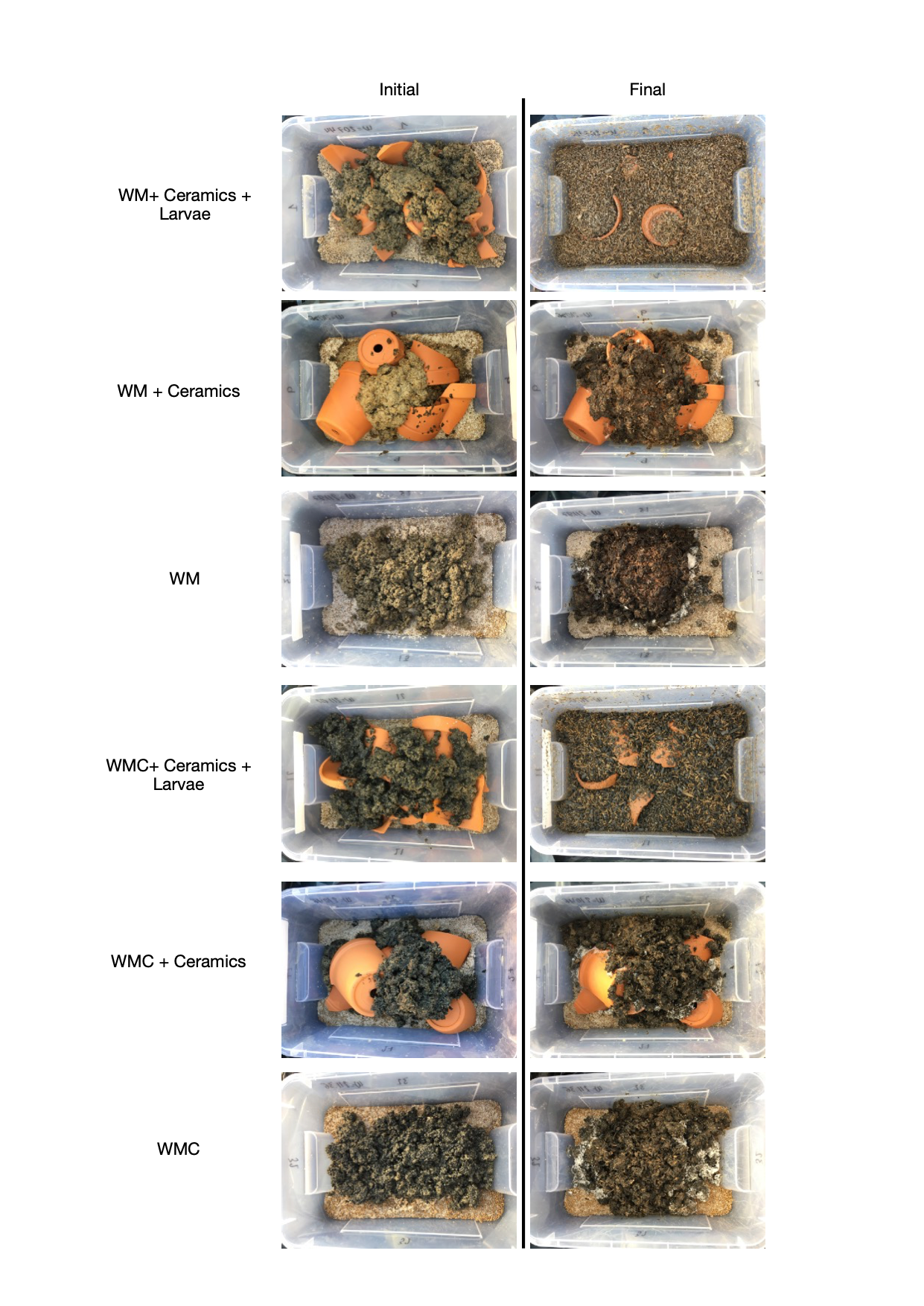


S2 Fig. BSFL’s ceramics burial and topsoil layer (faunal mantle) formation.

A pretty even soil layer is observed in the treatments with larvae whereas treatments that have only ceramics or waste remain at initial stages of waste decomposition.
