# Supplementary material for: Bioturbation by black soldier fly larvae—Rapid soil formation with burial of ceramic artifacts
Source: PLoS One. 2021 Jun 2;16(6):e0252032. doi: 10.1371/journal.pone.0252032 (PMC8171933; doi:10.1371/journal.pone.0252032)
Supplement: S3 Fig — BSF can be found distributed below altitudes of 2000 m (most of the Amazon basin). Photo: Juan M. Orozco-Ortiz. Leticia, Colombia. (DOCX) [file pone.0252032.s003.docx]

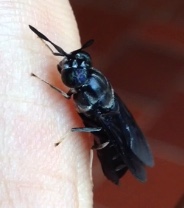


S3 Fig. Adult Black Soldier Fly (BSF) in the Amazon.

BSF can be found distributed below altitudes of 2000 m (most of the Amazon basin). Photo: Juan M. Orozco-Ortiz. Leticia, Colombia.
