# Supplementary material for: Bioturbation by black soldier fly larvae—Rapid soil formation with burial of ceramic artifacts
Source: PLoS One. 2021 Jun 2;16(6):e0252032. doi: 10.1371/journal.pone.0252032 (PMC8171933; doi:10.1371/journal.pone.0252032)
Supplement: S1 Table — (DOCX) [file pone.0252032.s004.docx]

| **S1 Table. Carbon (C) and Nitrogen (N) concentrations by layer** | | | | | | | | |
| --- | --- | --- | --- | --- | --- | --- | --- | --- |
| Treatments | | Layer | Thickness (cm) | N (g/kg^-1^) | | C (g/kg^-1^) | | C/N |
|  |  |  |  | mean | sd | mean | sd |  |
| Waste Model (WM) | Ceramics+BSFL | Organic Layer | 3.58±0.24 | 3.72 | 0.57 | 22.44 | 3.12 | 5.82 |
|  |  | Inorganic layer |  | 0.38 | 0.1 | 1.34 | 0.21 |  |
|  | Ceramics | Organic Layer | 1.88±0.4 | 15.51 | 2.13 | 136.62 | 15.96 | 8.72 |
|  |  | Inorganic layer |  | 0.32 | 0.06 | 1 | 0.12 |  |
|  | Control | Organic Layer | 1.86±0.17 | 12.02 | 3.57 | 109.82 | 30.96 | 8.99 |
|  |  | Inorganic layer |  | 0.34 | 0.04 | 1.14 | 0.11 |  |
| Waste Model with Charcoal (WMC) | Ceramics+BSFL | Organic Layer | 4.08±0.26 | 4.36 | 0.71 | 66.86 | 13.51 | 14.71 |
|  |  | Inorganic layer |  | 0.24 | 0.05 | 1.08 | 0.2 |  |
|  | Ceramics | Organic Layer | 2.32±0.32 | 17.05 | 6.38 | 209.38 | 84.26 | 12.04 |
|  |  | Inorganic layer |  | 0.3 | 0.09 | 1.28 | 0.33 |  |
|  | Control | Organic Layer | 1.76±0.6 | 18.01 | 4.23 | 217.04 | 45.89 | 11.94 |
|  |  | Inorganic layer |  | 0.36 | 0.07 | 1.38 | 0.13 |  |
| Soil Model |  | Total |  | 0.49 | 0.11 | 1.9 | 0.54 | 3.88 |
| Fresh WM |  |  |  | 47.49 | 7.23 | 400.3 | 16.05 |  |
| Fresh WMC |  |  |  | 42.57 | 8.54 | 457.9 | 14.96 |  |
